# Supplementary material for: Bioenergetic and Proteomic Profiling of Immune Cells in Myalgic Encephalomyelitis/Chronic Fatigue Syndrome Patients: An Exploratory Study
Source: Biomolecules. 2021 Jun 29;11(7):961. doi: 10.3390/biom11070961 (PMC8301912; doi:10.3390/biom11070961)
Supplement: Supplementary file 1 [file biomolecules-11-00961-s001.zip › Supplementary material 1.pdf]

ESM Materials and Methods

Table S1. Criteria for diagnosis of ME/CFS

Patient	Fukuda	Canada	ICC	IOM
**Patient 1**	Yes	Yes	Yes	Yes
**Patient 2**	Yes	Yes	Yes	Yes
**Patient 3**	Yes	No	Yes	Yes
**Patient 4**	Yes	Yes	Yes	Yes
**Patient 5**	Yes	Yes	Yes	Yes
**Patient 6**	Yes	No	No	No

Fukuda; Fukuda Criteria [5]
Canada: Revised Canada Criteria [49]
ICC: International Consensus Criteria [50]
IOM: Institute of Medicine [3]

A)

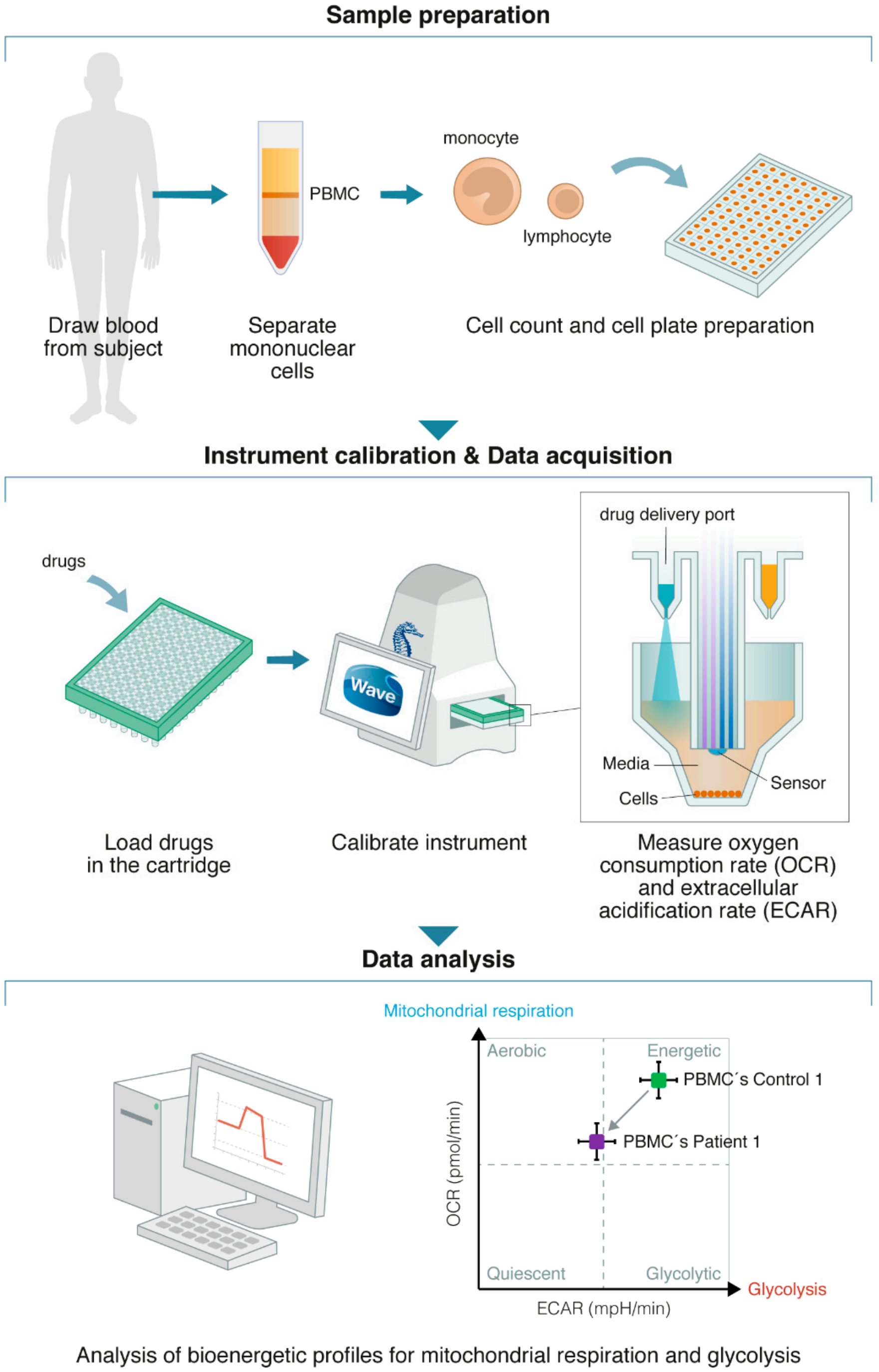

Sample preparation
PBMC
monocyte
lymphocyte
Draw blood from subject
Separate mononuclear cells
Cell count and cell plate preparation
Instrument calibration & Data acquisition
drugs
Wave
drug delivery port
Media
Cells
Sensor
Load drugs in the cartridge
Calibrate instrument
Measure oxygen consumption rate (OCR) and extracellular acidification rate (ECAR)
Data analysis
Mitochondrial respiration
Aerobic
Energetic
PBMC´s Control 1
PBMC´s Patient 1
Quiescent
Glycolytic
Glycolysis
OCR (pmol/min)
ECAR (mpH/min)
Analysis of bioenergetic profiles for mitochondrial respiration and glycolysis

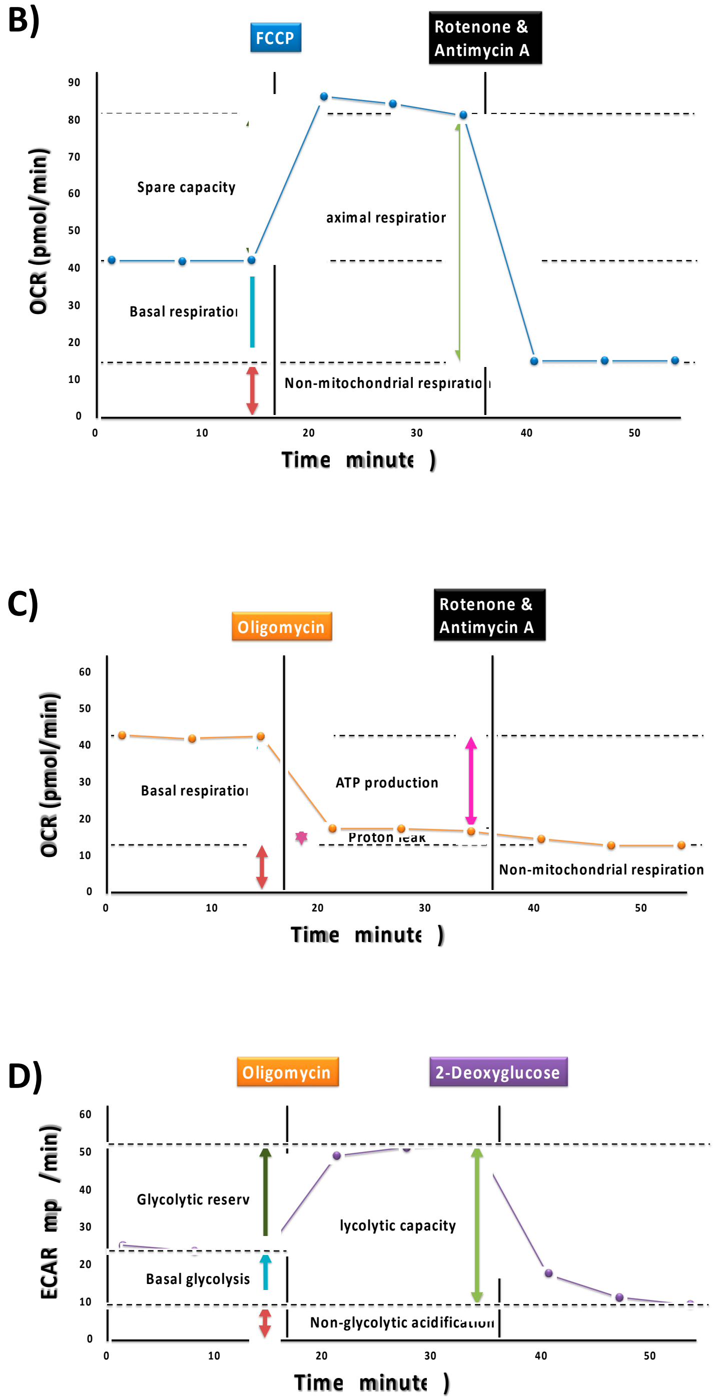

Figure. S1 Schematic representation of the bioenergetics profile analysis. (A) Diagram of the protocol used to perform the bioenergetics profile analysis **(B, C)** Diagram of the mitochondrial respiration analysis of PBMCs in the Seahorse XFe96. **(D)** Diagram of the non-mitochondrial glycolysis analysis of PBMCs in the Seahorse XFe96. The arrows indicate de values that were used to calculate the different parameters.

PBMCs: Peripheral Blood Mononuclear Cells. FCCP: Carbonyl cyanide 4-(trifluoromethoxy)phenylhydrazone. OCR: Oxygen Consumption Rate

ECAR: Extracellular Acidification Rate

Table S2. Equations and definitions of parameters from the bioenergetics profile

Parameter value	Equation	Definition
	**Mitochondrial related parameters (OCR)**	
**Non-mitochondrial oxygen consumption (pmol oxygen/min)**	Mean of the 3 OCR data points after Rotenone/Antimycin injection	Oxygen consumption from other sources than the respiratory chain such as NADH/NADPH oxidases. various desaturases and detoxification enzymes.
**Basal respiration (pmol oxygen/min)**	OCR values of the last 2 baseline data points before the first injection – non-mitochondrial oxygen consumption	Oxygen consumption used to meet cellular ATP demand reflecting the cellular energy demand under baseline conditions.
**Proton leak (pmol oxygen/min)**	OCR values of the 3 data points after oligomycin injection – non-mitochondrial oxygen consumption	Oxygen consumed with passive proton leakage across the mitochondrial inner membrane.
**ATP-linked respiration (pmol oxygen/min)**	Basal respiration – OCR values of the 3 data points after oligomycin injection	Oxygen consumed for ATP generation through complex V. Also called Oligomycin sensitive respiration (OSR).
**Maximal respiration (pmol oxygen/min)**	OCR values of the 3 data points after FCCP injection – non-mitochondrial oxygen consumption	Theoretical maximal respiration that a cell can achieve under increased energy demands mimicked by uncoupling the respiratory chain.
**Spare respiratory capacity (SRC)**	Maximal respiration / basal respiration	The ability of the cell to generate ATP via oxidative phosphorylation in response to increased energy demands. It can be an indicator of cell fitness or flexibility.
**Coupling efficiency (%)**	(ATP–linked respiration / basal respiration) x 100	Defines the fraction of protons used for mitochondrial ATP production proportional to protons leaking through the mitochondrial inner membrane. Higher coupling efficiency is indicative of mitochondria with high efficiency of substrate oxidation and ATP production.
**Cell Respiratory Control Ratio (RCR)**	Maximal respiration / proton leak	Determination of how much the mitochondrial membrane proton leak is tight to ATP synthesis. Higher RCR means mitochondria is more coupled to produce ATP.

		It can serve as a functional assessment of mitochondrial integrity. Denotes the capacity of mitochondria to oxidize respiratory substrates and to synthesize ATP
**ATP-linked to maximal respiration**	ATP – linked respiration / maximal respiration	Theoretical maximal ATP production capacity of the mitochondria without substrate or membrane potential limitations.
**Bioenegertic Health Index (BHI)**	(ATP – linked respiration * Spare respiratory capacity) / (Proton leak * non-mitochondrial respiration)	
	**Glycolysis related parameters (ECAR)**	
**Non-glycolytic acidification (mpH/min)**	ECAR values of the 3 data points after 2-DG injection	Other sources of extracellular acidification that are not attributed to lactate production by glycolysis such as acidification of carbon dioxide, a product of the TCA cycle and oxidative phosphorylation, which can be converted to bicarbonate generating protons at the same time.
**Basal glycolysis (mpH/min)**	ECAR of the last 2 baselines values before the first injection – non-glycolytic acidification	The process of producing lactate from glucose via the glycolysis pathway to meet cellular ATP demand under baseline conditions
**Maximal glycolytic capacity (mpH/min)**	ECAR values of the 3 data points after oligomycin injection	Theoretical maximum capacity of the cells to increase the anaerobic glycolysis rate in response to increases in energy demand
**Spare Glycolytic capacity (SGC) (mpH/min)**	Glycolytic capacity / basal glycolysis	Ratio of how close to the theoretical maximum are the cells using glycolysis. The ability of the cells to increase the anaerobic glycolysis rate in response to increases in energy demand. The cell's ability to respond to energy demands can be an indicator of cell fitness or flexibility.

OCR: Oxygen Consumption Rate

ECAR: Extracellular Acidification Rate

ESM. Targeted metabolomics profiling

Among the 408 metabolites, the AbsoluteIDQ® p400 kit (Biocrates Life Sciences AG, Innsbruck, Austria) allows the quantification of 55 acylcarnitines, 21 amino acids, 21 biogenic amines, 196 glycerophospholipids (24 lysophosphatidylcholines and 172 phosphatidylcholines), 40 sphingolipids (31 sphingomyelins and 9 ceramides), the sum of hexoses, 14 cholesteryl esters and 60 glycerides (18 diglycerides and 42 triglycerides). Subclasses of metabolites were named as follows: acylcarnitines (AC), lysophosphatidylcholines (LPC), phosphatidylcholines (PC), sphingomyelins (SM), ceramides (Cer), cholesteryl esters (CE), diglycerides (DG) and triglycerides (TG).

According to the Metabolomics Standards Initiative guidelines [1], metabolites are annotated with high confidence (level 1). However, it should be mentioned that for the FIA-HRMS-based metabolites, several isomers are quantified as one metabolite and further referred to as one metabolite. The extensive list of isomers provided by Biocrates is available upon request. Concentrations were reported in ng/mL plasma. Molecular weights used by the kit are available upon request.

The analysis was performed using a Combi PAL HTS TMO autosampler (Thermo Fisher Scientific, Waltham, MA, USA), an LX-2 LC system with two injectors (Thermo Fisher Scientific), each connected to an Ultimate 3000 Dionex RS pump and to the mass spectrometer through a Transcend II Valve Interface Module (Thermo Fisher Scientific). Mass spectrometry (MS) data were acquired on a QExactive (Thermo Fisher Scientific) with a heated electrospray ionization source.

When solvents were not provided with the kit, they were purchased from Thermo Fisher Scientific and of Optima™ LC/MS grade. The instruments were controlled using TraceFinder 4.1 Clinical Research, Aria MX V2.2 and QExactive tune software V2.8 SP1.

The 7-point calibration curve allows for absolute quantification of the LC-HRMS-based metabolites, whereas FIA-HRMS acquisition provides relative quantitative results based on 1-point calibrations (quantitative measurements for the 12 acylcarnitines).

LC-HRMS data was pre-processed using Xcalibur 4.1, integration of peaks was carefully checked, and all data were integrated into Biocrates MetIDQ Carbon-2793 software for quality assessment overview, quantification of metabolite concentrations by reference to internal standards, and normalization.

ESM. Steroid analysis

Solvents were LCMS-grade and purchased from Thermo Fischer Scientific (Waltham, MA, USA) or Sigma Aldrich (St. Louis, MO, USA). Ultrapure water (H_2O) was obtained on a Milli-Q IQ 7000 LC-Pak (Merck KGaA, Darmstadt, Germany). We used the targeted LCMS CHS MSMS Steroids Kit (PerkinElmer Inc., Waltham, MA, USA) to measure the concentration of 17-hydroxyprogesterone, testosterone, androstenedione and cortisol in the plasma samples. Each experimental sample was extracted once. We followed the manufacturer's instructions from sample preparation up to data processing. The instrument was an Acquity UPLC coupled to a Xevo TQ-S (Waters Corporation, Waltham, MA, USA). Target Lynx (Waters Corporation) was used to process the targeted LCMS data. Calibration and controls quality met the requirements defined by the manufacturer and by the Clinical and Laboratory Standards Institute (CLSI) guidelines C62-A, paragraph 7.3 [2]. For details, see supplementary data.

ESM Results

Table S3. Bivariate correlations demographics and questionnaires.

	FSCM	FSCM Mental fatigue	FSCM Physical fatigue	Compass-31	SF-36 Physical functioning	SF-36 Role limitation due to physical health	SF-36 Energy / fatigue	SF-36 Emotional well-being	SF-36 Social functioning	SF-36 Pain	SF-36 General health
Compass-31	0.93***	0.95***	0.89***								
SF-36 Physical functioning	-0.96***	-0.97***	-0.94***	-0.90***							
SF-36 Role limitation due to physical health	-0.92***	-0.87**	-0.95***	-0.73*	0.89***						
SF-36 Energy/fatigue	-0.84**	-0.88**	-0.80**	-0.86**	0.90***	0.70*					
SF-36 Emotional well-being	-0.72*	-0.77**	-0.67*	-0.82**	0.82**	0.59	0.91***				
SF-36 Social functioning	-0.88***	-0.92***	-0.84**	-0.87**	0.96***	0.75*	0.94***	0.86**			
SF-36 Pain	-0.75*	-0.67*	-0.81**	-0.50	0.68*	0.90***	0.54	0.35	0.52		
SF-36 General health	-0.87**	-0.86**	-0.86**	-0.81**	0.91***	0.83**	0.72*	0.73*	0.86**	0.62	
SF-36 Health change	0.07	-0.04	0.17	-0.26	0.09	-0.30	0.31	0.54	0.26	-0.56	0.08

*p-value < 0.05. ** p-value < 0.01. *** p-value < 0.001.

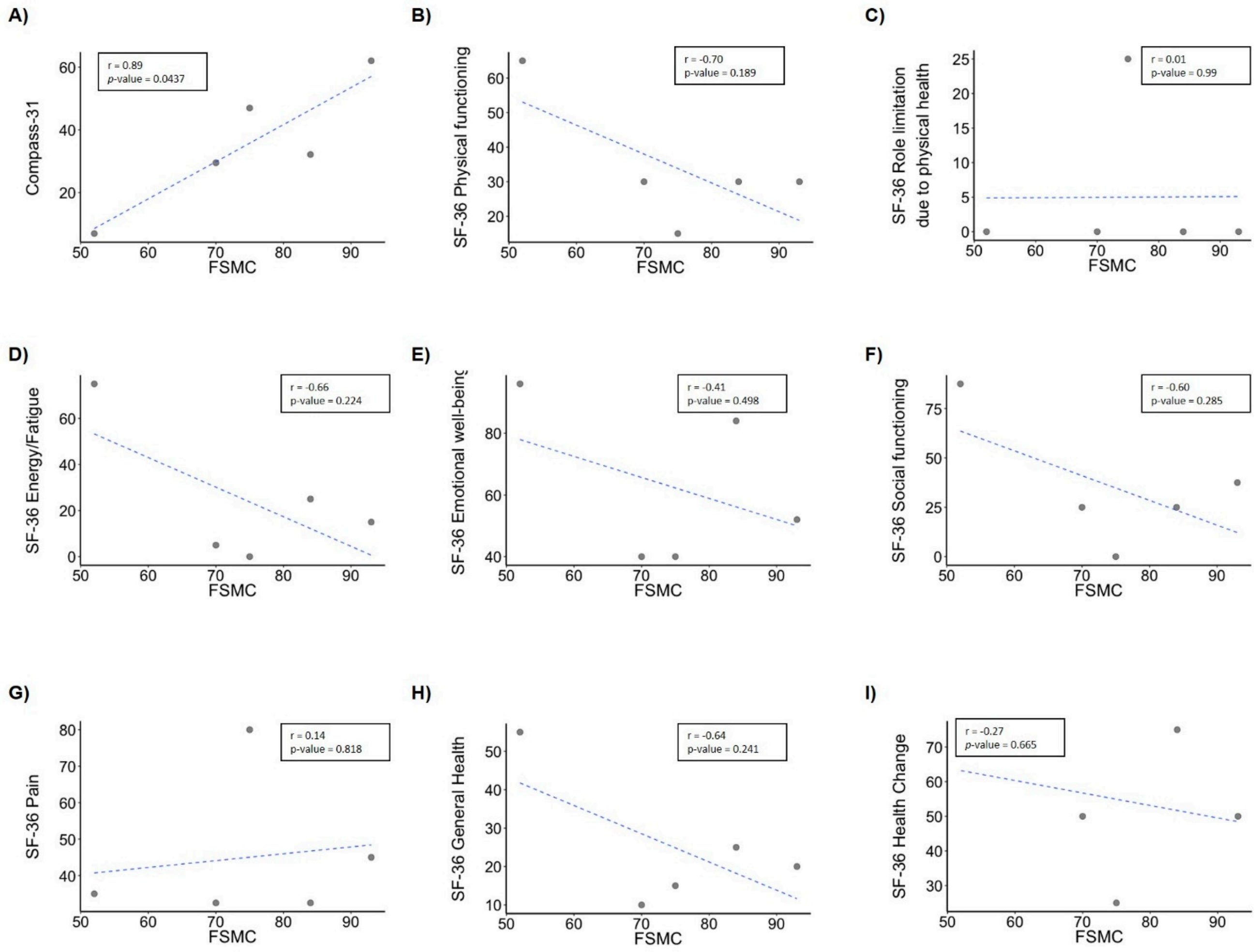

Figure. S2. Correlation analyses of self-assessed fatigue in ME/CFS patients. Pearson correlation analysis of FSCM total score with: **(a)** Compass-31, **(b)** SF-36 Physical functioning, **(c)** SF-36 Role limitation due to physical health, **(d)** SF-36 Energy and Fatigue, **(e)** SF-36 Emotional well-being, **(f)** SF-36 Social functioning, **(g)** SF-36 Pain, **(h)** SF-36 General Health, **(i)** SF-36 Health Change. *p*-values and Pearson (r) values were generated using Pearson's correlation analysis. n = 5 ME/CFS patients (data from one patient is missing due to lack of completion of questionnaires).

Table S4. Bioenergetics profile of PBMCs from controls and ME/CFS patients

Bioenergetics parameters	Controls (n = 4)	ME/CFS patients (n = 6)
**Basal respiration (pmol O_2/min)**	23.54 ± 4.39	21.91 ± 7.60
**Proton leak (pmol O_2/min)**	2.87 ± 1.29	5.67 ± 3.23
**ATP-linked respiration (pmol O_2/min)**	20.85 ± 3.91	18.17 ± 7.89
**Maximal respiration (pmol O_2/min)**	59.18 ± 9.94	66.32 ± 25.08
**Spare respiratory capacity**	2.61 ± 0.61	3.32 ± 0.37
**Non-mitochondrial respiration (pmol O_2/min)**	10.83 ± 3.28	10.17 ± 5.33
**Coupling efficiency (%)**	88.70 ± 4.65	**76.89 ± 10.20***
**Cell respiratory control ratio**	25.26 ± 15.87	13.67 ± 5.79
**ATP-linked to maximal respiration**	0.36 ± 0.08	0.27 ± 0.05
**Bioenergetics Health Index**	32.74 ± 28.71	21.44 ± 17.00
**Basal glycolysis (mpH/min)**	16.83 ± 6.65	18.20 ± 7.69
**Maximal glycolytic capacity (mpH/min)**	34.30 ± 6.21	37.43 ± 10.90
**Spare glycolytic capacity**	1.36 ± 0.78	1.42 ± 0.81
**Non-glycolytic acidification (mpH/min)**	9.21 ± 3.35	10.70 ± 7.81

Data are represented as mean ± SD
*p-value < 0.05

Table S5. Bivariate correlations questionnaires and bioenergetics profile

Characteristics	Disease duration	FSCM	FSCM Mental fatigue	FSCM Physical fatigue	Compass-31	SF-36 Physical functioning	SF-36 Role limitation due to physical health	SF-36 Energy / fatigue	SF-36 Emotional well-being	SF-36 Social functioning	SF-36 Pain	SF-36 General health
**Basal respiration (pmol O_2/min)**	-0.38	0	-0.04	0.03	-0.16	0.18	0.07	0.24	0.47	0.22	-0.12	0.19
**Proton leak (pmol O_2/min)**	0.50	0.62	0.57	0.66	0.49	-0.53	-0.62	-0.39	-0.40	-0.42	-0.60	-0.66
**ATP-linked respiration (pmol O_2/min)**	-0.40	0.01	-0.03	0.05	-0.12	0.19	0.07	0.21	0.45	0.22	-0.14	0.21
**Maximal respiration (pmol O_2/min)**	-0.10	0.35	0.33	0.37	0.18	-0.18	-0.24	-0.12	0.17	-0.18	-0.32	-0.11
**Spare respiratory capacity**	0.50	**0.76***	**0.78***	**0.73***	**0.78***	**-0.70***	-0.61	**-0.73***	-0.65	**-0.73***	-0.45	-0.59
**Non-mitochondrial respiration (pmol O_2/min)**	0.04	0.17	0.17	0.17	0.10	-0.09	-0.01	-0.20	-0.07	-0.16	-0.12	-0.10
**Coupling efficiency (%)**	**-0.84****	-0.63	-0.63	-0.63	-0.61	**0.73***	0.66	0.59	**0.77***	0.66	0.44	**0.86****
**Cell respiratory control ratio**	-0.47	-0.40	-0.36	-0.42	-0.34	0.42	0.46	0.21	0.35	0.28	0.35	0.59
**ATP-linked to maximal respiration**	-0.59	-0.58	-0.62	-0.53	-0.57	0.66	0.52	0.60	0.59	0.73	0.23	0.58
**Bioenergetics Health Index**	-0.38	-0.30	-0.31	-0.28	-0.36	0.36	0.18	0.32	0.44	0.35	0.06	0.50
**Basal glycolysis (mpH/min)**	0	0.16	0.14	0.18	0.36	-0.08	-0.08	-0.05	-0.24	-0.01	-0.06	-0.32

**Maximal glycolytic capacity (mpH/min)**	-0.06	0.45	0.42	0.47	0.41	-0.23	-0.28	-0.18	-0.06	-0.17	-0.32	-0.26
**Spare glycolytic capacity**	0.26	-0.02	-0.03	0.00	0.08	-0.08	-0.13	-0.12	-0.26	-0.04	-0.14	-0.10
**Non-glycolytic acidification (mpH/min)**	024	0.49	0.47	0.50	0.28	-0.38	-0.41	-0.32	-0.09	-0.38	-0.45	-0.34

*p-value < 0.05. ** p-value < 0.01. *** p-value < 0.001

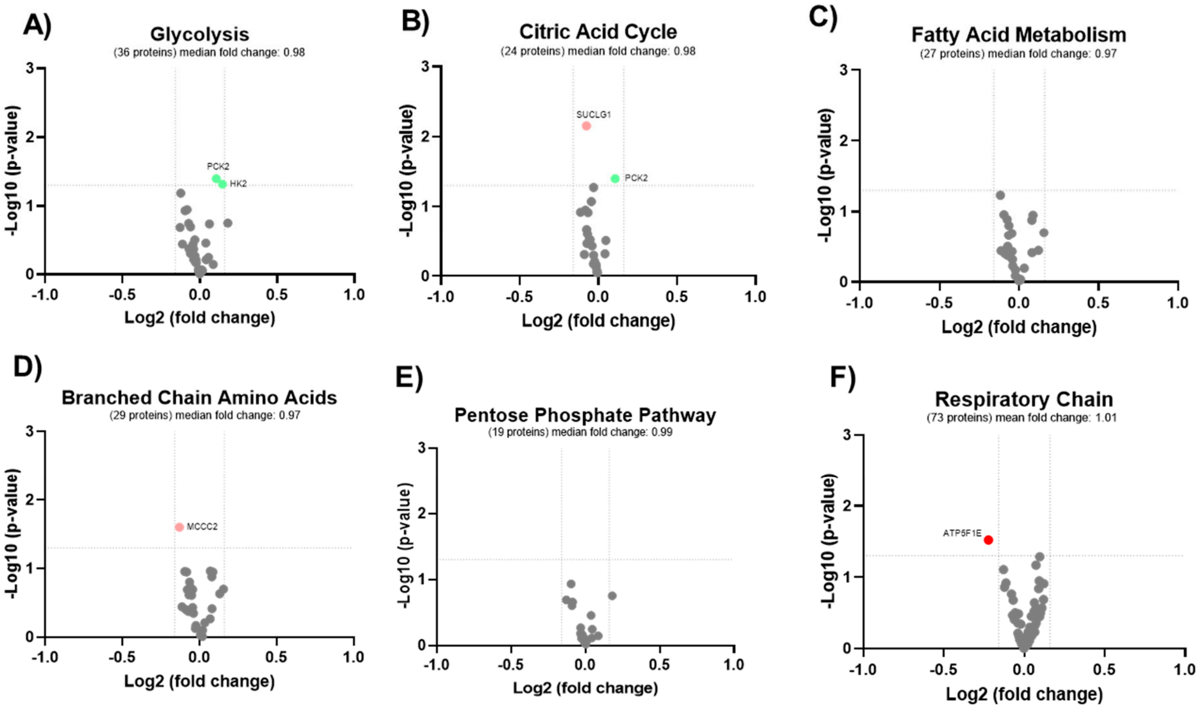

Figure. S3 Proteomics profile of central metabolic pathways of PBMCs from ME/CFS patients and healthy individuals as controls. Volcano plot of the proteins quantified belonging to (**A**) glycolysis, (**B**) citric acid cycle, (**C**) fatty acid metabolism, (**D**) branched-chain amino acid metabolism, (**E**) pentose phosphate pathway, and (**F)** respiratory chain. n = 10 (4 controls. 6 ME/CFS patients). Dark red/green dots denote significantly regulated proteins (passing both FC and p-value criteria). Light red/green dots denote proteins with significant p-value; however, not passing FC criteria.

Table S7. Steroid levels in plasma from controls and ME/CFS patients

Steroids levels	Controls (n = 4)	ME/CFS patients (n =6)	*p*-value
**17-Hydroxyprogesterone (nmol/L)**	1.73 ± 0.91	1.67 ± 1.64	0.944
**Testosterone (nmol/L)**	0.95 ± 0.33	0.77 ± 0.14	0.358
**Androstenedione (nmol/L)**	2.50 ± 0.74	2.60 ± 0.89	0.853
**Cortisol (nmol/L)**	205.03 ± 34.36	183.72 ± 43.87	0.42

References

1. Sumner, L.W.; Amberg, A.; Barrett, D.; Beale, M.H.; Beger, R.; Daykin, C.A.; Fan, T.W.-M.; Fiehn, O.; Goodacre, R.; Griffin, J.L.; et al. Proposed minimum reporting standards for chemical analysis. *Metabolomics* **2007**, *3*, 211–221, doi:10.1007/s11306-007-0082-2.
2. Clarke, W.; Molinaro, R.J.; Bachmann, L.M.; Botelho, J.C.; Cao, Z. *Liquid Chromatography-Mass Spectrometry Methods; Approved,* 1st ed.; Clinical and Laboratory Standards Institute: Wayne, PA, USA, 2014; Volume 34.
