# Supplementary material for: Bioenergetic and Proteomic Profiling of Immune Cells in Myalgic Encephalomyelitis/Chronic Fatigue Syndrome Patients: An Exploratory Study
Source: Biomolecules. 2021 Jun 29;11(7):961. doi: 10.3390/biom11070961 (PMC8301912; doi:10.3390/biom11070961)
Supplement: Supplementary file 1 [file biomolecules-11-00961-s001.zip › Electronic Supplementary Materials_MeTaQuaC.zip!/2020-12-16_ME-CFS_from_MeTaQuaC_to_MetaboAnalyst.html]

Targeted Metabolomics profiling of Myalgic Encephalomyelitis/Chronic Fatigue Syndrome patients and controls.


# Targeted Metabolomics profiling of Myalgic Encephalomyelitis/Chronic Fatigue Syndrome patients and controls.

### Statistical analyses using MetaboAnalystR3.0.3 as part of the publication by Fernandez-Guerra, Gonzalez-Ebsen, et al. 2021

#### Julie Courraud, PharmD, PhD (julc@ssi.dk)

#### Section for Clinical Mass Spectrometry, Danish Center for Neonatal Screening,

#### Department of Congenital Disorders, Statens Serum Institut Copenhagen, Artillerivej 5, 2300 Copenhagen S, Denmark

#### December 17, 2020

- Results
  - Data processing - Transformation and Scaling
  - Fold-change analysis
  - t-tests with false discovery rate (FDR) correction
  - Volcano plot and box plots
  - Principal Component Analysis
  - Hierarchical Clustering Heatmaps
- R script
  - 1- Formatting the compound concentrations table exported from the MeTaQuaC output into MetaboAnalyst format
  - 2- Performing transformation/scaling and statistical analyses using MetaboAnalystR3.03

# Results

## Data processing - Transformation and Scaling

The figure shows the compounds concentrations before and after generalized log (glog) transformation and Pareto scaling (see step 3.3 of the script).

Figure caption: Scaling results

## Fold-change analysis

The figure shows in purple the 25 compounds that are outside the defined *controls/patients* fold-change thresholds of: >1.3 or <0.77 (see step 4 of the script).

Figure caption: Fold-Change analysis

Below are shown the 25 compounds and their fold-change values.

## t-tests with false discovery rate (FDR) correction

The figure shows all p-values obtained from the univariate t-tests analyses (see step 5 of the script).

P-values are FDR-corrected.

No compound was found significantly different between patients and controls.

Figure caption: t-tests plots displaying p-values with FDR correction

Below is shown the list of all p-values before and after FDR correction.

## Volcano plot and box plots

The figure shows the volcano plot combining the controls/patients fold-change analysis to p-values *WITHOUT* FDR correction here (see step 6 of the script).

Two compounds have a p-value *before FDR correction* <0.05 and a fold-change (controls/patients) above 1.3: PC(36:4) and PC-O(34:4)

Figure caption: Volcano plot displaying p-values WITHOUT FDR correction

Below are shown the two selected compounds, their fold-change values and raw p-values.

Below are shown the two box plots displaying the selected compounds concentrations in patients and controls (see step 9 of the script).

## Principal Component Analysis

The PCA analysis reveals no separation of patients and controls (see step 7 of the script).

Figure caption: PCA scores plot

Figure caption: PCA scree plot

## Hierarchical Clustering Heatmaps

The first heatmap displays all compounds included int he analysis, while the second displays only the 10 compounds with lowest non-corrected p-value in the t-tests, i.e. <0.15 here (see step 8 of the script).

The heatmaps do not show a clear pattern differentiating patients and controls.

Figure caption: Heatmap displaying the selected 10 compounds

# R script

### 1- Formatting the compound concentrations table exported from the MeTaQuaC output into MetaboAnalyst format

Reading the exported tables

```
library(tidyverse)

ft_fia_all <- read.table ("C:/ME-CFS/ME-CFS_MeTaQuaC_exported_data_FIA.csv", sep=",", header=TRUE, dec=".", skip = 1, row.names = 1)
ft_lc_all <- read.table ("C:/ME-CFS/ME-CFS_MeTaQuaC_exported_data_LC.csv", sep=",", header=TRUE, dec=".", skip = 1, row.names = 1)
```

Combining sample measurements for compounds acquired with LC-HRMS and FIA-HRMS

```
ft_fia <- ft_fia_all[-c(1,2,3,4), ]
ft_fia <- select(ft_fia, starts_with("sample"))
ft_lc <- ft_lc_all[-c(1,2,3), ]
ft_lc <- select(ft_lc, starts_with("sample"))
ft <- rbind(ft_lc, ft_fia)
```

Transposing the dataframe to have compounds in columns and samples in rows.

```
ft <- as.data.frame(t(ft))
ft$Type <- gsub("Case", "Patients", ft$Type)
ft$Type <- gsub("Control", "Controls", ft$Type)
```

Making sure the classes are correct (“factor” for Type and “numeric” for compound concentrations to be able to use the ft directly)

```
for(i in c(2:ncol(ft))) {
  ft[,i] <- as.numeric(ft[,i])
}
ft[,1] <- as.factor(ft[,1])
sapply(ft, class)
```

```
##       Type        Ala        Arg        Asn        Cit        Gln        Glu 
##   "factor"  "numeric"  "numeric"  "numeric"  "numeric"  "numeric"  "numeric" 
##        Gly        His        Ile        Lys        Met        Orn        Phe 
##  "numeric"  "numeric"  "numeric"  "numeric"  "numeric"  "numeric"  "numeric" 
##        Pro        Ser        Thr        Trp        Tyr        Val       xLeu 
##  "numeric"  "numeric"  "numeric"  "numeric"  "numeric"  "numeric"  "numeric" 
##       ADMA Creatinine  Sarcosine       SDMA  Serotonin  t4-OH-Pro    Taurine 
##  "numeric"  "numeric"  "numeric"  "numeric"  "numeric"  "numeric"  "numeric" 
##    AC(0:0)    AC(2:0)   CE(16:1)   CE(18:2)   CE(18:3)   CE(20:4)   CE(20:5) 
##  "numeric"  "numeric"  "numeric"  "numeric"  "numeric"  "numeric"  "numeric" 
##   CE(22:5)   CE(22:6)   DG(34:1)   DG(36:2)   DG(36:3)   DG(36:4)   DG(39:0) 
##  "numeric"  "numeric"  "numeric"  "numeric"  "numeric"  "numeric"  "numeric" 
##   DG(41:1)   TG(48:1)   TG(48:2)   TG(50:1)   TG(50:2)   TG(50:3)   TG(50:4) 
##  "numeric"  "numeric"  "numeric"  "numeric"  "numeric"  "numeric"  "numeric" 
##   TG(51:2)   TG(51:3)   TG(51:4)   TG(52:2)   TG(52:3)   TG(52:4)   TG(52:6) 
##  "numeric"  "numeric"  "numeric"  "numeric"  "numeric"  "numeric"  "numeric" 
##   TG(53:4)   TG(53:5)   TG(54:3)   TG(54:4)   TG(54:5)   TG(54:6)   TG(56:6) 
##  "numeric"  "numeric"  "numeric"  "numeric"  "numeric"  "numeric"  "numeric" 
##   TG(56:7)   TG(56:8)  LPC(16:0)  LPC(16:1)  LPC(18:0)  LPC(18:1)  LPC(18:2) 
##  "numeric"  "numeric"  "numeric"  "numeric"  "numeric"  "numeric"  "numeric" 
##  LPC(20:4)  LPC(22:6) PC-O(34:1) PC-O(34:2) PC-O(34:3) PC-O(34:4) PC-O(35:3) 
##  "numeric"  "numeric"  "numeric"  "numeric"  "numeric"  "numeric"  "numeric" 
## PC-O(36:2) PC-O(36:3) PC-O(36:4) PC-O(36:5) PC-O(38:4) PC-O(38:5) PC-O(38:6) 
##  "numeric"  "numeric"  "numeric"  "numeric"  "numeric"  "numeric"  "numeric" 
## PC-O(40:4) PC-O(40:5) PC-O(40:6) PC-O(42:4) PC-O(42:5) PC-O(42:6) PC-O(44:6) 
##  "numeric"  "numeric"  "numeric"  "numeric"  "numeric"  "numeric"  "numeric" 
##   PC(29:0)   PC(30:0)   PC(32:0)   PC(32:1)   PC(32:2)   PC(33:0)   PC(33:1) 
##  "numeric"  "numeric"  "numeric"  "numeric"  "numeric"  "numeric"  "numeric" 
##   PC(33:4)   PC(34:1)   PC(34:3)   PC(34:4)   PC(35:1)   PC(35:2)   PC(35:3) 
##  "numeric"  "numeric"  "numeric"  "numeric"  "numeric"  "numeric"  "numeric" 
##   PC(35:4)   PC(36:2)   PC(36:3)   PC(36:4)   PC(37:1)   PC(37:2)   PC(37:4) 
##  "numeric"  "numeric"  "numeric"  "numeric"  "numeric"  "numeric"  "numeric" 
##   PC(37:5)   PC(37:6)   PC(38:0)   PC(38:1)   PC(38:3)   PC(38:4)   PC(38:5) 
##  "numeric"  "numeric"  "numeric"  "numeric"  "numeric"  "numeric"  "numeric" 
##   PC(38:6)   PC(39:3)   PC(39:5)   PC(39:6)   PC(40:2)   PC(41:4)   PC(42:7) 
##  "numeric"  "numeric"  "numeric"  "numeric"  "numeric"  "numeric"  "numeric" 
##   PC(43:6)   PC(44:1)   PC(44:3)  Cer(42:1)  Cer(42:2)   SM(32:1)   SM(32:2) 
##  "numeric"  "numeric"  "numeric"  "numeric"  "numeric"  "numeric"  "numeric" 
##   SM(33:1)   SM(33:2)   SM(34:1)   SM(34:2)   SM(35:1)   SM(36:1)   SM(36:2) 
##  "numeric"  "numeric"  "numeric"  "numeric"  "numeric"  "numeric"  "numeric" 
##   SM(37:1)   SM(38:1)   SM(38:2)   SM(39:1)   SM(39:2)   SM(40:2)   SM(41:1) 
##  "numeric"  "numeric"  "numeric"  "numeric"  "numeric"  "numeric"  "numeric" 
##   SM(41:2)   SM(42:1)   SM(42:2)   SM(42:3)   SM(43:1)   SM(43:2)   SM(44:1) 
##  "numeric"  "numeric"  "numeric"  "numeric"  "numeric"  "numeric"  "numeric"
```

Creating the CSV file that will be used by MetaboAnalystR

```
write.table(ft, 'C:/ME-CFS/ME-CFS_MeTaQuaC_exported_data.csv', sep=',',col.names = NA, row.names = TRUE, quote = TRUE, na="NA")
```

### 2- Performing transformation/scaling and statistical analyses using MetaboAnalystR3.03

See the guide at https://www.metaboanalyst.ca/docs/RTutorial.xhtml

```
library(amap)
library(RJSONIO)
library("MetaboAnalystR")
packageVersion("MetaboAnalystR")
```

```
## [1] '3.0.3'
```

#### STEP 1: Creating the mSet Object, specifying that the data to be uploaded is a compound concentration table (“conc”) and that statistical analysis will be performed (“stat”)

```
mSet<-InitDataObjects("conc", "stat", FALSE)
```

```
## Starting Rserve...
##  "C:\Users\julc\DOCUME~1\R\WIN-LI~1\4.0\Rserve\libs\x64\Rserve.exe" --no-save 
## [1] "MetaboAnalyst R objects initialized ..."
```

#### STEP 2: Reading in the concentration table, please set the path right first

```
mSet<-Read.TextData(mSet, "C:/ME-CFS/ME-CFS_MeTaQuaC_exported_data.csv", "rowu", "disc")
```

#### STEP 3: Performing data processing using MetaboAnalystR (filtering/normalization)

3.1: Performing data processing - Data checking

```
mSet<-SanityCheckData(mSet)
```

```
##  [1] "Successfully passed sanity check!"                                                                                
##  [2] "Samples are not paired."                                                                                          
##  [3] "2 groups were detected in samples."                                                                               
##  [4] "Only English letters, numbers, underscore, hyphen and forward slash (/) are allowed."                             
##  [5] "<font color=\"orange\">Other special characters or punctuations (if any) will be stripped off.</font>"            
##  [6] "All data values are numeric."                                                                                     
##  [7] "A total of 19 (1.2%) missing values were detected."                                                               
##  [8] "<u>By default, missing values will be replaced by 1/5 of min positive values of their corresponding variables</u>"
##  [9] "Click the <b>Skip</b> button if you accept the default practice;"                                                 
## [10] "Or click the <b>Missing value imputation</b> to use other methods."
```

3.2: Performing data processing - Minimum Value Replacing

Missing values will be replaced by 1/5 of min positive values of their corresponding variables

```
mSet<-ReplaceMin(mSet)
```

3.3: Performing data processing - Transformation and Scaling

“Normalization” in MetaboAnalyst implies “row-wise” normalization, which is sample-based: we don’t apply any.

Then transformation (generalized log).

Then column-wise scaling (feature-based) including Pareto.

Pareto scaling is mean-centered and divided by the square root of the SD

```
mSet<-PreparePrenormData(mSet)
mSet<-Normalization(mSet, "NULL", "LogNorm", "ParetoNorm", ratio=FALSE, ratioNum=20)
```

Visualizing (saving the figures in your work directory)

```
mSet<-PlotNormSummary(mSet, "norm_0_", "png", 300, width=NA)
mSet<-PlotSampleNormSummary(mSet, "snorm_0_", "png", 300, width=NA)
```

Saving your tables in your working directory

```
mSet<-SaveTransformedData(mSet)
```

#### STEP 4: Performing fold-change analysis

```
mSet<-FC.Anal.unpaired(mSet, 1.3, 0)
mSet<-PlotFC(mSet, "fc_0_", "png", 300, width=NA)
```

#### STEP 5: Performing t-test analysis (with FDR correction)

```
mSet <- Ttests.Anal(mSet, FALSE, 0.1, FALSE, TRUE, TRUE)
```

```
## [1] "A total of 0 significant features were found."
```

```
mSet <- PlotTT(mSet, "tt_0_", "png", 300, width=NA)
```

#### STEP 6: Creating a volcano plot combining the fold-change analysis to p-values WITHOUT FDR correction here.

```
mSet<-Volcano.Anal(mSet, FALSE, 1.3, 0, 0.75,F, 0.05, TRUE, "raw")
mSet<-PlotVolcano(mSet, "volcano_0_",1, "png", 300, width=NA)
```

#### STEP 7: Performing Principal Component Analysis (PCA)

```
mSet<-PCA.Anal(mSet)
mSet<-PlotPCAPairSummary(mSet, "pca_pair_0_", "png", 300, width=NA, 5)
mSet<-PlotPCAScree(mSet, "pca_scree_0_", "png", 300, width=NA, 5)
mSet<-PlotPCA2DScore(mSet, "pca_score2d_0_", "png", 300, width=NA, 1,2,0.95,0,0)
mSet<-PlotPCALoading(mSet, "pca_loading_0_", "png", 300, width=NA, 1,2);
mSet<-PlotPCABiplot(mSet, "pca_biplot_0_", "png", 300, width=NA, 1,2)
mSet<-PlotPCA3DLoading(mSet, "pca_loading3d_0_", "json", 1,2,3)
```

#### STEP 8: Visualizing data in Hierarchical Clustering Heatmaps.

8.1: full list of compounds

```
mSet<-PlotHeatMap(mSet, "heatmap_0_", "png", 300, width=NA, "norm", "row", "euclidean", "complete","bwm", "overview", T, T, NULL, T, F)
```

8.2: displaying only the 10 compounds with lowest p-values without FDR correction in the t-tests, i.e. here <0.15.

```
mSet<-PlotSubHeatMap(mSet, "heatmap_10comp_", "png", 300, width=NA, "norm", "row", "euclidean", "complete","bwm", "tanova", 10, "overview", T, T, T, F)
```

#### STEP 9: Creating individual boxplots for visualization of selected compounds and export as pdf

```
pdf(file="PC364_PC-O344_boxplots.pdf", width=10, height=5)

p1 <- ggplot2::ggplot(ft, ggplot2::aes(x = Type, y = ft[,109], color = Type)) +
    ggplot2::geom_boxplot(outlier.shape = NA) +
    ggplot2::geom_jitter(shape = 16, position = position_jitter(0.2)) +
    ggplot2::labs(y = "Concentrations in ng/mL") +
    ggplot2::labs(title = "PC(36:4)") +
    ggplot2::scale_x_discrete(labels = c(Controls = "Controls", Patients = "Patients")) +
    ggplot2::scale_colour_manual(values = c(rgb(96, 108, 158, maxColorValue = 255),
                                            rgb(193, 88, 88, maxColorValue = 255))) +
    ggplot2::theme_bw() +
    ggplot2::theme(panel.border = element_blank(), 
                   panel.grid.major = element_blank(),
                   panel.grid.minor = element_blank(), 
                   axis.line = element_line(colour = "black", size = 0.75),
                   axis.title.x = element_blank(),
                   axis.title.y = element_text(size = 20, 
                                               margin = margin(t = 0, r = 20, b = 0, l = 0)),
                   axis.text = element_text(colour = "black", size = 18),
                   plot.title = element_text(hjust = 0.5, size = 20),
                   legend.position = "none")

p2 <- ggplot2::ggplot(ft, ggplot2::aes(x = Type, y = ft[,76], color = Type)) +
    ggplot2::geom_boxplot(outlier.shape = NA) +
    ggplot2::geom_jitter(shape = 16, position = position_jitter(0.2)) +
    ggplot2::labs(y = "Concentrations in ng/mL") +
    ggplot2::labs(title = "PC-O(34:4)") +
    ggplot2::scale_x_discrete(labels = c(Controls = "Controls", Patients = "Patients")) +
    ggplot2::scale_colour_manual(values = c(rgb(96, 108, 158, maxColorValue = 255),
                                            rgb(193, 88, 88, maxColorValue = 255))) +
    ggplot2::theme_bw() +
    ggplot2::theme(panel.border = element_blank(), 
                   panel.grid.major = element_blank(),
                   panel.grid.minor = element_blank(), 
                   axis.line = element_line(colour = "black", size = 0.75),
                   axis.title.x = element_blank(),
                   axis.title.y = element_text(size = 20, 
                                               margin = margin(t = 0, r = 20, b = 0, l = 0)),
                   axis.text = element_text(colour = "black", size = 18),
                   plot.title = element_text(hjust = 0.5, size = 20),
                   legend.position = "none")

grid.arrange(p1, p2, nrow = 1)

dev.off()
```

```
## png 
##   2
```
